# Effects of Anesthesia and Surgery on the Morphologic and Functional Development of the Premature Neonatal Brain: A Systematic Review and Meta-Analysis

**DOI:** 10.3390/jcm14030918

**Published:** 2025-01-30

**Authors:** Annalena G. U. Heisel, Markus F. Stevens, Marsh Königs, Faridi S. Jamaludin, Kristin Keunen, Jorinde A. W. Polderman

**Affiliations:** 1 Department of Anesthesiology, Location AMC, Amsterdam University Medical Centres, P.O. Box 22660, 1100 DD Amsterdam, The Netherlands; 2 Department of Pediatrics, Emma Children’s Hospital, Amsterdam UMC Location University of Amsterdam, Meibergdreef 9, 1105 AZ Amsterdam, The Netherlands; 3 Amsterdam Reproduction and Development Research Institute, Meibergdreef 9, 1105 AZ Amsterdam, The Netherlands; 4 Amsterdam UMC Location University of Amsterdam, Medical Library AMC, Meibergdreef 9, 1105 AZ Amsterdam, The Netherlands

**Keywords:** brain, development, anesthesia, surgery, infants, neonatal

## Abstract

**Background:** The percentage of preterm infants requiring surgery before 44 weeks of postmenstrual age (PMA) varies between 19% and 36%. The potential impact of general anesthesia on the vulnerable developing brain of preterm infants remains unknown. **Methods:** A systematic review and meta-analysis on the impact of general anesthesia on brain integrity and neurodevelopmental outcomes in preterm infants undergoing surgery before 44 weeks PMA was conducted. Studies were identified via a PubMed, EMBASE (Ovid), and Cochrane CENTRAL search conducted from inception until 8 March 2023, following PRISMA guidelines. Brain abnormality was assessed using MRI-based brain volume and abnormality scores. Neurodevelopment was evaluated through Bayley Infant and Toddler Development (BSID) or Wechsler Preschool and Primary Scale of Intelligence (WPPSI) tests. Quality was assessed via the Cochrane ROBINS-I tool and GRADE. **Results:** Our systematic search identified 2883 records, leading to the inclusion of 12 observational studies. Very low-quality evidence suggests that preterm infants exposed to anesthesia were more likely to show postoperative brain abnormalities on MRI (OR 2.01, 95%CI 1.24–3.25, *p* = 0.005). They had lower neurodevelopmental scores on the BSID II and III (psychomotor developmental index: mean difference (MD) −10.98; 95%CI −12.04 to −9.91; *p* < 0.001 and cognitive composite score: (MD) −10.11; 95%CI −11.06 to −9.16; *p* < 0.001 at two years of age compared to preterm infants not exposed to anesthesia. **Conclusion:** Exposure to surgery and anesthesia before term age is associated with brain abnormalities and neurodevelopmental delay at two years, but conclusions are limited by low evidence quality, uncontrolled confounders, and the methodological biases of the included studies; thus further robust studies are required (PROSPERO:CRD42021255907).

## 1. Background

The percentage of preterm infants who require surgery before term-equivalent age, i.e., before 44 weeks of postmenstrual age (PMA), varies between 19% and 36% [1,2]. Abundant preclinical studies have shown that anesthetic agents have toxic effects on the brain cells of neonatal animals [3]. Subsequent retrospective clinical studies have revealed that early exposure to anesthesia could potentially result in enduring cognitive and learning deficits [4,5]. In 2016, the U.S. Food and Drug Administration (FDA) issued a Drug Safety Communication addressing the possible neurotoxic impact of anesthesia in pediatric patients. The FDA conveyed that the “repeated or lengthy use of general anesthetic and sedation drugs during surgeries or procedures in children younger than 3 years or in pregnant women during their third trimester may affect the development of children’s brains” [6].

Subsequent to the FDA cautionary notice, three prospective landmark studies, including the Pediatric Anesthesia Neuro Development Assessment (PANDA) study [7], the Mayo Anesthesia Safety in Kids (MASK) study [8], and the General Anesthesia or Awake-regional Anesthesia in Infancy (GAS) trial [9], demonstrated that a brief and single exposure to general anesthesia during early childhood does not affect cognitive and motor development in otherwise healthy children. Preterm infants exposed to anesthesia before 44 weeks PMA were not included in these studies.

The choice of 44 weeks PMA as a threshold is chosen because of its critical role in brain development. Prior to 44 weeks PMA, the brain undergoes significant growth and maturation, including the development and integration of neural networks, such as the thalamocortical circuitry, which forms at between 24 and 44 weeks PMA [10]. This phase is also characterized by rapid cortical sulcation, synaptogenesis, myelination, cerebellar growth, neuronal maturation, axonal development, and a peak in microglial abundance, all of which are essential for normal neurodevelopment [11,12]. These intricate and dynamic processes make the developing brain particularly vulnerable to external influences during this critical window. The findings of Gano et al. further emphasize the importance of this period to 44 weeks, demonstrating that exposure to anesthesia for surgery prior to term-equivalent age (TEA) is associated with significantly reduced cognitive performance in cases with multiple exposures, whereas exposures occurring after TEA do not exhibit this association [11]. These observations highlight the sensitivity of the pre-TEA period to potential disruptions and underscore the need for further research into the impact of anesthetic exposure and surgical interventions on brain development during this time, as during this period, anesthesia is often required for congenital malformations or the complications of prematurity [13].

When exposed to general anesthesia in this crucial period of neurodevelopment, the trajectories of brain development may be disrupted [14]. The perioperative mechanisms leading to postoperative neurodegeneration are heterogeneous. Keunen et al. proposed that a combination of and interaction between inflammation, immature vascularization combined with perioperative hemodynamic instability and neurotoxicity of anesthetic drugs may result in a multiform picture of brain injury, such as punctate white matter injury or stroke [10]. Inflammation can be systemic, caused by the underlying disease (e.g., necrotizing enterocolitis (NEC)) and worsened by surgical stress [10]. Immature vascularization in the brain involves underdeveloped autoregulation and the incomplete growth of cerebral vessels, which can be further compromised by abnormal CO_2_ levels or drugs during anesthesia [10]. Moreover, most sedative drugs routinely used during anesthesia can trigger the loss of neurons through apoptosis [15].

Several studies investigated the relationship between general anesthesia exposure in early life, brain abnormality scores and neurodevelopment outcome later in life. Limited evidence suggests that brain abnormalities visualized by magnetic resonance imaging (MRI) might be able to predict worse neurodevelopmental outcomes later in life [16]. Smaller brain volumes correlate with worse motor performance and neurodevelopmental outcome at 24 months and with a lower mental developmental scale at age 3.5 years [17].

The primary aim of this systematic review and meta-analysis is to summarize the existing evidence on the association between general anesthesia exposure during surgery in preterm infants before 44 weeks PMA, brain abnormalities measured with MRI, and neurodevelopmental outcomes. 

## 2. Methods

The protocol of this systematic review and meta-analysis was registered in PROSPERO (CRD42021255907) and conducted according to the Meta-analysis Of Observational Studies in Epidemiology (MOOSE) guidelines [18].

### 2.1. Systematic Literature Search

For our study, we used the Preferred Reporting Items for Systematic Reviews (PRISMA) guidelines. We systematically searched the electronic databases PubMed, EMBASE (Ovid) and Cochrane Central Register of Controlled Trials (CENTRAL) from inception through to the 8th of March 2023 to identify randomized controlled trials and prospective and retrospective observational cohort studies that included control subjects who had not undergone surgery. We included the following terms and their equivalents into our search strategy, which was designed by a medical librarian: neonates or preterm infants, surgery or anesthesia, and neurodevelopment (tests) or MRI. The full search PubMed strategy can be found in Supplementary Appendix S1. Neither time nor language restrictions were applied. To retrieve full data from conference papers, we contacted authors twice via email to request additional data.

### 2.2. Study Selection

After deduplication, the identified records were imported in Rayyan for screening [19]. Two researchers (J.P. and A.H.) independently screened titles and abstracts. Disagreements were resolved by discussion. Full text screening was carried out by the same two researchers. Disagreements were again resolved by discussion. When no agreement was found, a third reviewer (M.S.) was consulted. In cases of overlapping study populations, the individual reports were analyzed as one study.

We selected studies with the following: (1) a cohort of preterm infants born at < 37 weeks PMA who underwent surgery under general anesthesia before 44 weeks PMA, hereafter known as the exposed group, and (2) a control group of preterm infants who did not undergo surgery and received no general anesthesia before 44 weeks PMA, hereafter known as the non-exposed group. Studies were included if they covered the following: (3) a cerebral MRI scan, neurodevelopmental outcome, or intelligence outcome. We excluded cardiac surgery, neurosurgery, and neonates with genetic disorders.

Among the included studies, the following data were extracted: (1) brain volumetry on MRI (total brain volume, white matter, deep nuclear gray matter, cortical gray matter); (2) brain abnormalities on MRI, scored with a systematic scoring system; and (3) neurodevelopment and intelligence outcomes assessed with the Bayley Scales of Infant Development (BSID) and/or the Wechsler Preschool and Primary Scale of Intelligence (WPPSI).

The BSID II provides indices for cognitive development (Mental Developmental Index (MDI)) and motor development (Psychomotor Developmental Index (PDI)) [20]. The Bayley-III consists of three domains, namely the Cognitive Composite Score (CCS), Motor Composite Score (MCS), and Language Composite Score (LCS) [21]. The WPPSI is the most widely utilized instrument for assessing intelligence in children aged 2.5 years and older. The Full Scale Intelligence Quotient (FSIQ) represents a comprehensive measure of cognitive ability derived from assessments such as the WPPSI [22,23].

### 2.3. Data Extraction

Data extraction from the relevant studies was performed independently by two authors (J.P. and A.H.) using a predefined data collection form. For each study, we extracted year of publication, study design, study population size, country, period of inclusion, type of surgery, mean GA (weeks), mean GA at exposure anesthesia (surgery), and the age at MRI or/and neurodevelopmental outcome assessment. We also searched for other moments of anesthesia exposure apart from surgery and explored whether anesthesia was also applied during the MRI.

### 2.4. Quality Assessment

Two authors (J.P. and A.H.) independently assessed the risk of bias using the Cochrane ROBINS-I tool [24]. This tool identifies four domains, namely I: failure to develop and apply appropriate eligibility criteria; II: flawed measurement of both exposure and outcome, III: failure to adequately control confounding; and IV: incomplete or inadequately short follow-up [25]. Studies were scored as ‘high-risk’ in the first category when eligibility criteria were not clearly prescribed and a high risk for selection bias was present in cases. In category II, studies were scored as high-risk when, in order to achieve outcome measurements, the standardized tests BSID or WPPSI were applied by an untrained professional or MRI scans were evaluated by less than two independently trained assessors. In category III, studies were scored as high-risk if anesthesia was used for MRI scanning, if data on possible confounding factors like prematurity and socioeconomic level were unavailable, and if the control group underwent anesthesia. In category IV, studies were scored as ‘high-risk’ in cases of missing data or a >30% loss to follow up. Inconsistencies were resolved by discussion and if necessary, a third author (M.S.) was consulted. The GRADE approach was used to determine the certainty of evidence per outcome measure. The summary of findings can be found in Supplementary Appendix S4 [26].

### 2.5. Statistical Analysis

We used the Review Manager (version 5.4) software for this meta-analysis. For the continuous data of the volumetry and BSID outcomes, the mean difference (MD) with 95% CI between the exposed and non-exposed groups was calculated. Brain abnormality scores were dichotomized. Any abnormality was scored as abnormalities present. For this outcome, we calculated the odds ratio (OR) with 95% CI. We performed one subgroup analysis comparing premature infants with NEC stage III (surgical NEC) to infants with NEC stage IIB or lower (non-surgical NEC) in order to minimize confounding by the disease itself. We considered a *X*^2^ test *p* value of < 0.05 and an I^2^ statistic value of >60% as cut-offs for statistical heterogeneity [27]. When statistical heterogeneity was identified, we explored possible causes. If unexplained, we proceeded with the meta-analysis using a random-effects model.

## 3. Results

### 3.1. Identification of Relevant Studies

**Figure 1:**
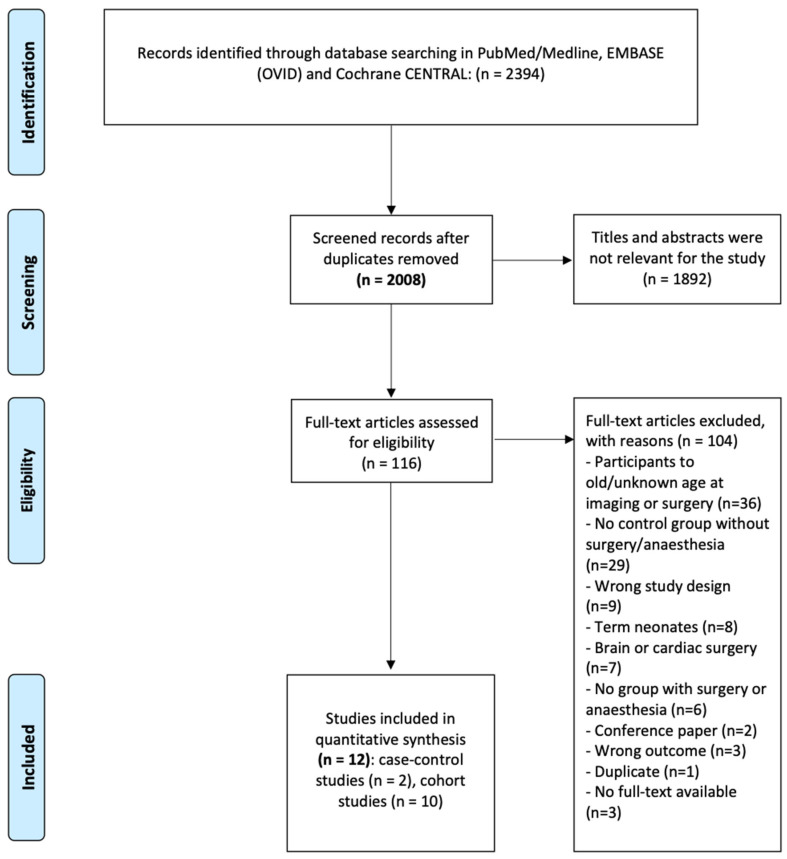
Selection process for studies included in the meta-analysis according to the PRISMA 2009 flow diagram.

Our search identified 2883 records from inception until the 8th of March 2023. After deduplication, we screened 2008 records based on title and abstract and identified 116 articles for full text screening. After screening the full texts, we included 12 studies for the qualitative analyses (Figure 1).

### 3.2. Study Characteristics

Included studies all had an observational design: ten cohort-studies and two case–control studies. Eight studies had prospectively collected data (Table 1). In total, we included 3376 preterm infants exposed to surgery with general anesthesia and 24,457 preterm infants not exposed to surgery with general anesthesia. The majority of studies reported on gastro-intestinal surgeries ([2,28,29,30,31,32,33,34,35,36], Table 1).

Five studies performed MRI scans in exposed and non-exposed preterm infants [2,11,28,29,30]. The MRI scans were performed at term-equivalent age. To calculate brain volumes, different segmentation techniques were used (Table 1). Segmentation techniques for MRI scans differed significantly. The segmentation technique used by Filan et al. was not able to distinguish deep nuclear gray matter from cortical gray matter and incorrectly included a part of the cerebellum when analyzing deep nuclear gray matter and cortical gray matter [2]. Therefore, we chose not to pool the deep nuclear gray matter, cortical gray matter, and total brain volume from Filan et al. as this could lead to misleading results.

Five different brain abnormality scores for MRI were used by the included studies [37,38,39,40]. The scoring systems of Inder and Woodward et al. systematically assessed the nature and extent of white matter injury, loss of periventricular white matter volume, cystic abnormalities, corpus callosum thinning, and lateral ventricle volume [2,30], whereas the scoring system of Kidokoro also included the cortical gray matter, deep gray matter, and cerebellar abnormalities [28,29]. Gano et al. also used the scoring system of Papile to systematically assess intracranial hemorrhage.

Seven studies evaluated neurodevelopment using the BSID II, and three studies used the BSID III (Table 1). All studies assessed the BSID in patients between 18 and 26 months of age. One study assessed intelligence using the WPPSI in patients at five years of age [11].

**Table 1:** Study characteristics.

Study	Prospective/Retrospective	Inclusion Period	Included Patients Exp vs. Non-Exp.	Mean Age at Birth (wks.)	Mean Age at Surgery	Type of Surgery	MRI: Timing and Analyses	BSID II/III or WPPSI
BSID II/III and MRI								
Filan et al. 2012 [2]	Prospective cohort study	2001–2004	*n* = 30 vs. *n* = 178	27.1 vs. 27.6	33.4 (26–41) wks.	Inguinal hernia, bowel, PDA closure	MRI < TEA Quant: Warfield Qual: Inder	BSID II at 24 months corrected age
Kojima et al. 2023 [28]	Prospective cohort study	2016–2019	*n* = 45 vs. *n* = 347	27.0 vs. 30.0	Before MRI scan	Gastro-intestinal, pulmonary, PDA closure, ROP laser	MRI: ±43 wks. Quant: dHCP Qual: Kidokoro	BSID III at 24 months corrected age
Walsh et al. 2020 [29]	Retrospective analysis of prospective cohort study	2007–2010	*n* = 25 vs. *n* = 59	25 vs. 27	27 (26–32) wks.	Gastro-intestinal, inguinal hernia, PDA closure, ROP laser	MRI: 37–38 wks. Quant: ANTs with ITK Snap Qual: Kidokoro	BSID III at 18–24 months corrected age
WPSII and MRI								
Gano et al. 2015 [11]	Prospective cohort study	1998–2009	*n* = 43 vs. *n* = 94	27.9	<42 weeks PMA	Laparotomy and PDA closure	MRI < TEA Qual: Miller and Papile Qant: -	WPPSI III between three and six years corrected age
MRI								
Garg et al. 2023 [30]	Retrospective cohort study	2013–2020	*n* = 48 vs. *n* = 59	26 vs. 28	13 (5–25) days	NEC	MRI: < TEA Quant: - Qual: Woodward	-
BSID II								
Allendorf et al. 2018 [31]	Retrospective case–control study	2006–2013	*n* = 24 vs. *n* = 13	27 + 6 vs. 27.4	Not reported	NEC	-	BSID II at 24 months corrected age
Dilli et al. 2012 [32]	Prospective case–control	2007–2009	*n* = 6 vs. *n* = 40	28.5	Not reported	NEC	-	BSID II at 18–24 months corrected age
Hintz et al. 2005 [33]	Retrospective cohort study	1995–1998	*n* = 124 vs. *n* = 121	83% birth < 28 wks.	Not reported	NEC	-	BSID II at 18–22 months corrected age
Shah et al. 2012 [34]	Retrospective analyses of prospective collected data	1998–2009	*n* = 32 vs. *n* = 785	25.7 vs. 26.2	Not reported	NEC	-	BSID II at 18–22 months corrected age
BSID II and III								
Bell et al. 2021 [35]	Retrospective analyses of prospective collected data	2008–2016	*n* = 364 vs. *n* = 4557	24.5 vs. 25.0	Not reported	NEC or SIP	-	BSID III at 18–26 months corrected age
Fullerton et al. 2017 [36]	Retrospective analysis of prospective collected data	1999–2012	*n* = 449 vs. *n* = 9063	25 vs. 26	Not reported	NEC	-	BSID II and III at 18–24 months corrected age
Morriss et al. 2014 [41]	Retrospective cohort study	1998–2009	*n* = 2186 vs. *n* = 9141	99.4% vs. 97.6% born < 30 wks.	Not reported	All types of surgery	-	BSID II and III at 18–22 months corrected age

Abbreviations: ANTs: Advanced Normalization Tools; BSID: Bayley scales of infant and toddler development; dHCP: developing Human Connectome Pipeline software; Exp: exposed; FAST: FMRIB Automated Segmentation Tool; NEC: necrotic enterocolitis; non-exp: not exposed; PDA: persistent ductus arteriosus; ROP: retinopathy of prematurity; TEA: term-equivalent age; Quant: quantitative; Qual: qualitative; vs.: versus; wks.: weeks; WPPSI: Wechsler preschool and primary scale of intelligence. Refs: Warfield et al. [42]; Walsh et al. [29] used Advanced Normalization Tools software with manual correction using ITK Snap software tools [43]. Kojima et al. [28] used the developing Human Connectome Pipeline software [44].

### 3.3. Quality Assessment

Details about our assessment of the potential risk of bias in the included studies are summarized in Supplementary Appendix S2. The study by Dili et al. did not have clear inclusion or exclusion criteria and was therefore scored as having a high risk of bias in the first domain [32]. Six out of twelve studies had an unknown risk of bias due to missing information on the assessors of the BSID assessment or on the number of (independent) assessors of the MRI. Seven out of twelve included studies were scored as ‘high-risk’ in category III due to uncontrolled confounding. In two studies, it was unclear whether patients received anesthesia for the MRI or surgery for any other condition [31,34].

### 3.4. Brain Volumetry (MRI)

**Figure 2:**
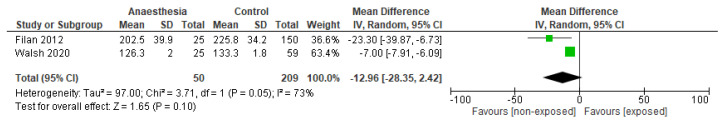
Effect of anesthesia exposure on white matter volume in neonates, compared to the non-exposed group [2,29].

Four studies (*n* = 791) assessed brain volumes in exposed and non-exposed preterm infants (2, 28–30). White matter volume was reported in two studies [2,29]. White matter volume was not significantly smaller in exposed preterm infants (*n* = 55) compared to non-exposed preterm infants (*n* = 237) (MD −12.96 cm^3^; 95%CI −28.35–2.42; *p* = 0.10; Figure 2). The certainty of evidence was downgraded to very low due to risk of bias (confounding), imprecision (small studies), and inconsistency (I^2^ = 73%).

### 3.5. Brain Abnormality Score (MRI)

**Figure 3:**
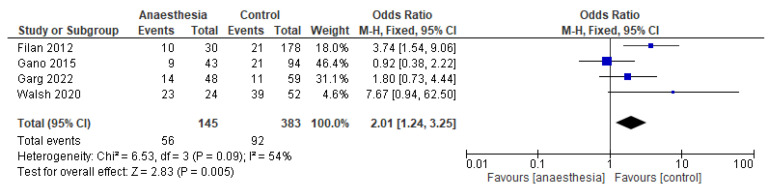
Effect of anesthesia exposure on brain abnormalities in neonates, compared to the non-exposed group [2,11,29,30].

Five studies assessed brain abnormalities using a systematic scoring system [2,11,28,29,30]. Brain abnormality scores were dichotomized. Any abnormality was scored as abnormalities present. Pooled results of four studies showed higher odds for brain abnormalities in exposed preterm infants compared to non-exposed preterm infants (OR 2.01; 95%CI 1.24–3.25; *p* = 0.005; Figure 3). The certainty of evidence was downgraded to very low due to imprecision (small studies, different scoring systems) and risk of bias (two out of five studies scored high on risk of bias, Supplementary Appendix S2). The fifth study did not report absolute numbers of brain abnormality scores but found that exposure to surgery with general anesthesia was associated with higher brain abnormality scores after propensity score matching. Matching included baseline, maternal, and neonatal characteristics significantly associated with both exposure to surgery and outcome [28]. 

### 3.6. Neurodevelopmental Outcome Score (WPPSI or BSID)

**Figure 4:**
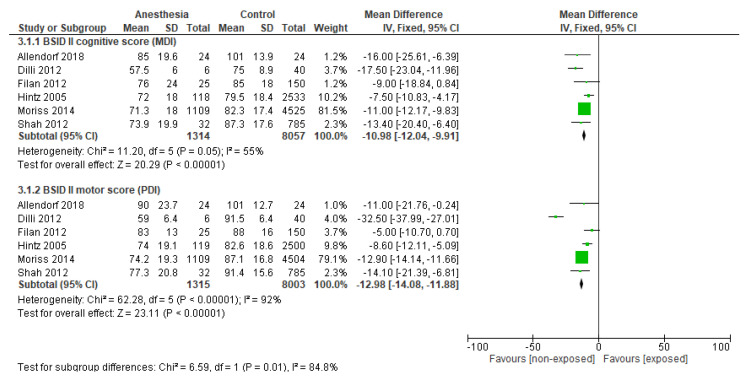
Effect of anesthesia exposure on neurodevelopmental outcome measured with BSID II in neonates, compared to the non-exposed group. Abbreviations: MDI: Mental Developmental Index; PDI: Psychomotor Developmental Index [2,29,31,32,33,34,41].

The meta-analysis on BSID II outcome scores included six studies (Figure 4). This analysis showed that exposed preterm infants (*n* = 1315) had lower BSID II scores at 18–24 months compared to non-exposed preterm infants (*n* = 8057) (MDI: MD −10.98, 95%CI −12.04 to −9.91, *p* < 0.001; PDI: MD −12.98, 95%CI −14.08 to −11.88, *p* < 0.001; Figure 4). The certainty of evidence was downgraded to very low due to the high risk of bias and inconsistencies in the majority of the studies (MDI: I^2^ = 55%; PDI: I^2^ = 92%). 

**Figure 5:**
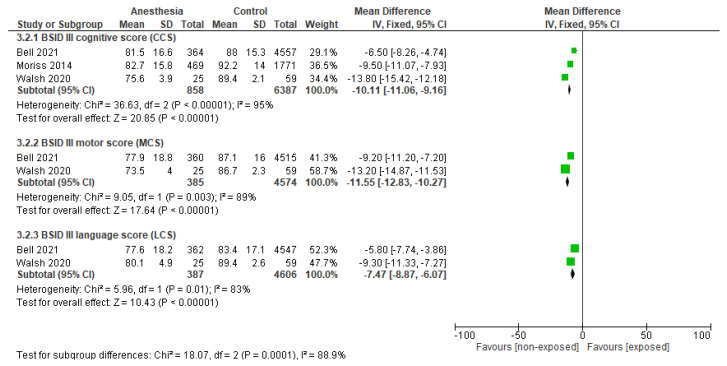
Effect of anesthesia on neurodevelopmental outcome measured with BSID III in neonates, compared to non-exposed. Abbreviations: CCS: Cognitive Composite Score; LCS: Language Composite Score; MCS: Motor Composite Score [29,35,41].

The meta-analysis on BSID III outcome scores included three studies (Figure 5). This analysis showed that exposed preterm infants (*n* = 858) had lower BSID III scores at 18–26 months compared to non-exposed preterm infants (*n* = 6387) (CCS: MD −10.11, 95%CI −11.06 to −9.16, *p* < 0.001, three studies; MCS: MD −7.47, 95%CI −8.87 to −6.07, *p* < 0.001, two studies; LCS: MD −11.55, 95%CI −12.83 to −10.27, *p* < 0.001, two studies; Figure 5). The certainty of evidence was downgraded to very low due to a high risk of bias and moderate inconsistencies (I^2^ ≥83%) in three out of four studies.

One study investigated the WPPSI scores after 4.6 years. A single surgery before term-equivalent age (TEA) and surgery after TEA were not associated with a significant difference in IQ-scores after correction for confounding factors, such as gestational age, prenatal steroids, hypotension, persistent ductus arteriosus, NEC, number of infections, duration of mechanical ventilation, white matter injury, and number of MRI under sedation. Exposure to two or more surgeries before TEA was associated with lower full-scale IQ after correction for confounding factors (MD −20.3, 95%CI −32.6 to −10.1). These results remained statistically significant after controlling for gestational age [11].

### 3.7. Subgroup Analysis in Preterm Infants with NEC

Seven studies specifically reported on neurodevelopmental outcomes in preterm infants with NEC [30,31,32,33,34,35,36]. Four studies also reported neurodevelopmental scores for preterm infants with NEC stage IIb and lower (non-surgical NEC) compared to preterm infants with NEC stage III and higher (surgical NEC) [31,32,33,34].

**Figure 6:**
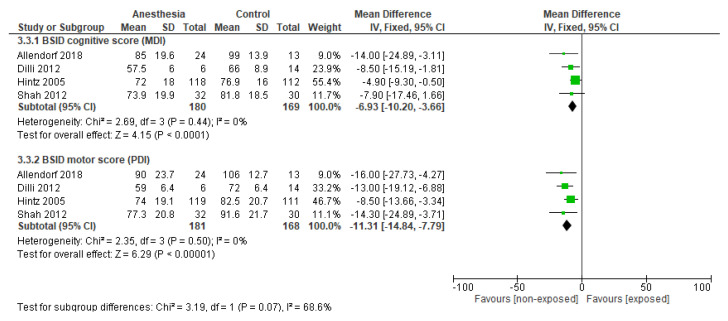
Subgroup analysis of neurodevelopmental outcome in neonates with surgical NEC compared to neonates with non-surgical NEC. Abbreviations: MDI: Mental Developmental Index; PDI: Psychomotor Developmental Index [31,32,33,34].

Preterm infants with a non-surgical NEC served as the control group in this meta-analysis to compare the effects of anesthesia. Four studies were included in the meta-analysis [31,32,33,34]. Preterm infants with surgical NEC scored significantly lower on the BSID II compared to preterm infants with non-surgical NEC (PDI: MD −6.93, 95%CI −10.20 to −3.66, *p* < 0.001; MDI: MD −11.31, 95%CI −14.84 to −7.79, *p* < 0.001; Figure 6). The certainty of evidence was very low due to imprecision (small sample size) and risk of bias [35,36].

Garg et al. found that 14 (43.8%) out of 32 preterm infants with surgical NEC had white matter injury as seen via MRI, compared to the preterm with non-surgical NEC of which 11 (23.4%) out of 59 preterm infants had white matter injury [30].

### 3.8. Publication Bias

The funnel plots for brain abnormalities, according to BSID II and BSID III, showed asymmetry, indicating possible publication bias in favor of studies with positive results (Supplementary Appendix S3). 

## 4. Discussion

The pooled results of this meta-analysis suggest that surgery with general anesthesia in preterm infants at <44 weeks PMA is associated with more brain abnormalities via MRI and lower neurodevelopmental outcome scores on the BSID II and III scales at around two years of age when compared to preterm infants not exposed to surgery with general anesthesia. The influence of confounding factors was poorly controlled in nearly all studies. 

Only one study performed extensive propensity matching for prominent confounders [28]. This study found an association between higher global brain abnormality scores on MRI and lower neurodevelopmental outcome scores at two years of age, in line with previous studies [45,46,47]. However, they did not find an association between infants exposed to anesthesia and lower neurodevelopmental outcome scores [28]. Kojima et al. interpreted this lack of an association in their study as the possibility that the study was underpowered and the BSID neurodevelopmental outcome scores may be less sensitive than the MRI. Also, brain abnormalities seen via MRI do not always correlate with clinical outcomes. Additionally, a prospective cohort study in extremely preterm infants found that major surgery was associated with brain injury seen in MRI at TEA, even when corrected for gestational age and days of mechanical ventilation. The authors only found an association with lower motor scores on the BSID at 18 months after correction for gestational age at birth and clinical illness score. No associations were found in language and cognitive scores [48]. Unfortunately, it is unknown whether the brain abnormalities found in the postoperative MRI scans were caused by the anesthesia needed for surgery or if they might have had a different etiology, as none of the studies included in the meta-analysis had a preoperative MRI scan as a baseline comparison. In our meta-analysis, the pooling of dichotomized data from different brain injury scoring systems on MRI could have led to overestimating the effect, as every abnormality was scored as abnormal.

Neurodevelopmental outcome is influenced by many factors. A major confounding factor in the assessment of morphologic brain development and neurodevelopment is premature birth itself, as this is also associated with smaller brain tissue volumes and developmental delay compared to healthy infants born at term; therefore, preterm infants without surgical exposure were chosen as the control group [49]. Disease prior to surgery can also alter normal brain development [10,49]. To control for the bias of the underlying illness, we performed a subgroup analysis in preterm infants diagnosed with NEC. We compared infants who required surgery for NEC with preterm infants who did not require surgery, although the severity of NEC still remains a confounding factor. In this analysis, we found that preterm infants with surgical NEC scored significantly lower on the BSID II compared to those with non-surgical NEC, with very low certainty of evidence due to imprecision and risk of bias. Finally, one should keep in mind that the majority of the included studies did not control for confounding factors like socio-economic status, gender, complications during pregnancy or childbirth, comorbidities like bronchopulmonary dysplasia, antenatal steroid use, and the type of care facility, so the results of this meta-analysis should be interpreted with caution [50]. 

The BSID is a widely used assessment tool for neurodevelopment after preterm birth. However, the prognostic value of the BSID scores is limited [51]. Thus, although our meta-analysis showed that exposure to anesthesia was associated with significantly lower BSID scores around two years of age, these scores could represent a lag in development instead of a permanent delay. The only study that used the WPPSI at five years of age found no difference in intelligence, [11] comparable to the GAS study, which also showed no difference in intelligence five years after a single anesthesia exposure after term equivalent age [9,52]. A side note must be made on the number of times anesthesia was required. Gano et al. found lower IQ scores at 4.6 years of age in the children who had two or more surgeries before TEA, after accounting for gestational age and illness severity [11]. Similar results were found in a cohort study by Flick et al., who found that multiple surgeries before the age of two independently increased the risk of learning disabilities later in life [5].

Another possibility is that negative outcomes after anesthesia before 44 weeks PMA may manifest as behavioral problems instead of poor cognition. Three large prospective studies, including the previously mentioned GAS trial, did not find an effect of anesthesia on neurodevelopmental outcome [8,9,52]. The pooled analyses of these trials also showed no effect on IQ at ages 5–20 years. However, parent-reported behavioral problems were significantly more prevalent in the group exposed to anesthesia [53], indicating that at the very least, we have to include behavioral assessments in the outcome parameters of future studies. Unfortunately, the Child Behavior Checklist or other behavioral assessments were not reported in the included studies of this meta-analysis.

As mentioned before, the findings of this systematic review and meta-analysis must be interpreted with caution. Seven of the twelve included studies had a high risk of bias as they lacked adequate control for confounding factors, limiting our results. Furthermore, as baseline MRIs are lacking, it is unknown if anesthesia played a causal role in the development of brain abnormalities seen on MRI if no baseline MRI is available. Nonetheless, to our knowledge, this is the first meta-analysis which summarizes the available MRI data and neurodevelopmental outcome data for preterm infants who required surgery with general anesthesia before 44 weeks PMA.

## 5. Conclusions and Future Perspectives

The evidence so far suggests that exposure to surgery with general anesthesia before 44 weeks PMA is associated with more brain abnormalities seen on MRI at term equivalent age and lower BSID scores at two years of age. A single exposure was not associated with lower intelligence scores at five years of age. The data should be interpreted with caution because the number of studies is limited and at high risk of bias. Preterm infants belong to our most fragile, but also most resilient, patient populations and the long-term effects of these findings remain to be elucidated. A role for future research is to investigate the possible causal role of anesthesia on the postoperative brain abnormalities found on MRI, ideally by performing preoperative MRI scans in preterm neonates. Furthermore, we advocate for the incorporation of behavioral assessment and long-term follow-up to investigate the effects of early-life surgery and anesthesia on behavioral domains and to better understand their long-term implications. 

## Data Availability

Data will be made available on request.

## Supplementary Materials

The following supporting information can be downloaded at: https://www.mdpi.com/article/10.3390/jcm14030918/s1, Appendix S1: PubMed search strategy; Appendix S2: Results of quality assessment of the observational studies using ROBIN-1 tool of cochrane [25]; Appendix S3: Publication bias funnel plot; Appendix S4: Summary of findings; Appendix S5: PRISMA 2020 checklist.

## Abbreviations

BSID	Bayley Scales of Infant Development
CCS	Cognitive Composite Score
FDA	U.S. Food and Drug Administration
FSIQ	Full Scale Intelligence Quotient
GAS	General Anesthesia or Awake-regional Anesthesia in Infancy trial
LCS	Language Composite Score
MASK	Mayo Anesthesia Safety in Kids study
MCS	Motor Composite Score
MD	mean difference
MDI	Mental Developmental Index
MOOSE	Meta-analysis Of Observational Studies in Epidemiology guidelines
MRI	magnetic resonance imaging
NEC	necrotizing enterocolitis
OR	odds ratio
PANDA	Pediatric Anesthesia Neuro Development Assessment study
PDI	Psychomotor Developmental Index
PMA	postmenstrual age
PRISMA	Preferred Reporting Items for Systematic Reviews guidelines
TEA	term-equivalent age
WPPSI	Wechsler Preschool and Primary Scale of Intelligence
