# Supplementary material for: Effects of Anesthesia and Surgery on the Morphologic and Functional Development of the Premature Neonatal Brain: A Systematic Review and Meta-Analysis
Source: J Clin Med. 2025 Jan 30;14(3):918. doi: 10.3390/jcm14030918 (PMC11818576; doi:10.3390/jcm14030918)
Supplement: Supplementary file 1 [file jcm-14-00918-s001.zip › jcm-3383187-supplementary.pdf]

Supplementary File S1

Appendix S1. PubMed Search Strategy

((“Brain/growth and development”[Mesh] OR “Nervous System/growth and development”[Mesh] OR “Neurodevelopmental Disorders”[Mesh] OR “Child Development”[Majr] OR “Mental Processes/growth and development”[Mesh] OR neurodevelopment*[tiab] OR brain[tiab] OR intellectual developmental disorder*[tiab] OR behavioural disorder*[tiab] OR behavioral disorder*[tiab] OR behavior disorder*[tiab] OR behaviour disorder*[tiab])

AND

(“Magnetic Resonance Imaging”[Mesh] OR magnetic resonance imag*[tiab] OR MR imag*[tiab] OR MRI[tiab] OR MRIs[tiab] OR Wechsler[tiab] OR WPPSI[tiab] OR Bayley[tiab] OR BSID[tiab])

AND

(“Infant, Newborn”[Mesh] OR neonat*[tiab] OR infant*[tiab] OR newborn*[tiab] OR prematur*[tiab] OR preterm*[tiab] OR fetus[tiab] OR fetal[tiab])

AND

(“Surgical Procedures, Operative”[Mesh] OR “Anesthesia”[Mesh] OR anesthe*[tiab] OR anaesthe*[tiab] OR surg*[tiab] OR operat*[tiab]))

NOT

(“Letter”[Publication Type] OR “Editorial”[Publication Type] OR “Comment”[Publication Type] OR “Case Reports”[Publication Type] OR “Review”[Publication Type] OR “Letter”[Title] OR “Editorial”[Title] OR “case report”[Title])

NOT

((“Animals”[MeSH Terms] OR “Animal Experimentation”[MeSH Terms] OR “animals, laboratory”[MeSH Terms] OR “models, animal”[MeSH Terms] OR “animal*”[Title/Abstract] OR “rat”[Title/Abstract] OR “rats”[Title/Abstract] OR “mice”[Title/Abstract] OR “mouse”[Title/Abstract] OR “dog”[Title/Abstract] OR “dogs”[Title/Abstract] OR “pig”[Title/Abstract] OR “pigs”[Title/Abstract] OR “cow”[Title/Abstract] OR “cows”[Title/Abstract] OR “monkey”[Title/Abstract] OR “monkeys”[Title/Abstract] OR “horse”[Title/Abstract] OR “horses”[Title/Abstract] OR sheep[tiab] OR ovine[tiab] OR lamb[tiab] OR lambs[tiab] OR goat*[tiab] OR swine[tiab] OR porcine[tiab] OR pup[tiab] OR pups[tiab] OR canine[tiab] OR bitch*[tiab] OR beagle[tiab] OR feline[tiab] OR rodent*[tiab] OR rabbit*[tiab] OR murine[tiab] OR ape[tiab] OR apes[tiab] OR gorilla[tiab] OR gorillas[tiab] OR “catfish”[Title/Abstract]) NOT “Humans”[MeSH Terms])

Appendix S2. Results of Quality Assessment of the Observational Studies Using ROBIN-1 Tool of Cochrane [25].

	I	II	III	IV
Allendorf, 2018 [31]				
Bell, 2021 [35]				
Dilli, 2012 [32]				
Filan, 2012 [2]				
Fullerton, 2017 [36]				
Gano, 2015 [11]				
Garg, 2022 [30]				
Hintz, 2005 [33]				
Kojima, 2022 [28]				

Morriss, 2014 [41]	green	yellow	yellow	green
Shah, 2012 [34]	green	green	yellow	green
Walsh, 2020 [29]	green	yellow	green	green

I: Failure to develop and apply appropriate eligibility criteria (inclusion of control population); II: flawed measurement of both exposure and outcome; III: failure to adequately control confounding; IV: incomplete or inadequately short follow-up. Color codes: green—low risk; red—high risk; yellow—unclear.

Appendix S3. Publication Bias Funnel Plot

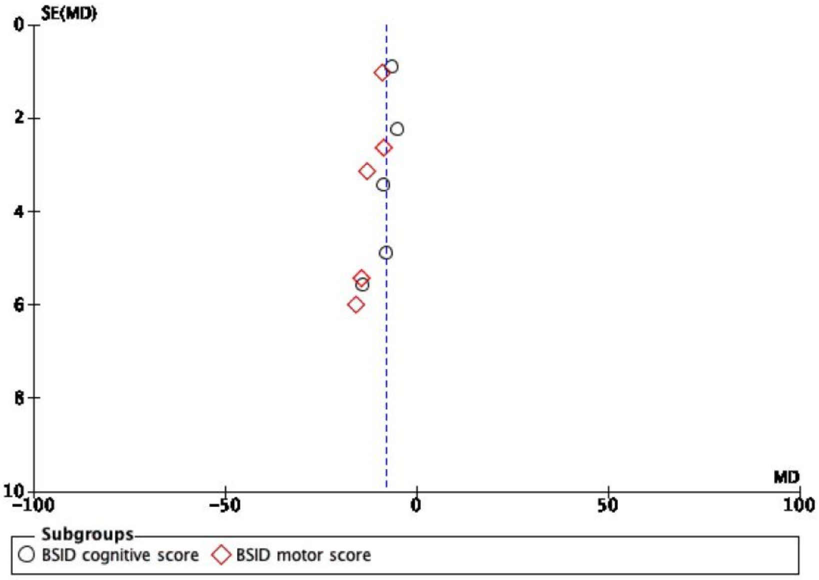

Figure S1. Funnel plot of BSID subgroup analysis in infants with necrotizing enterocolitis (NEC). Abbreviations: BSID: Bayley Scales of Infant and Toddler Development.

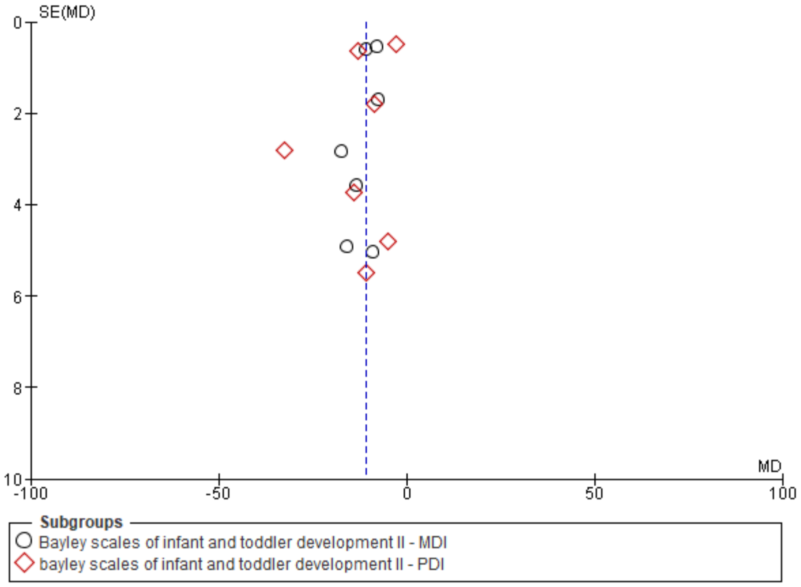

Figure S2. Funnel plot of BSID II.

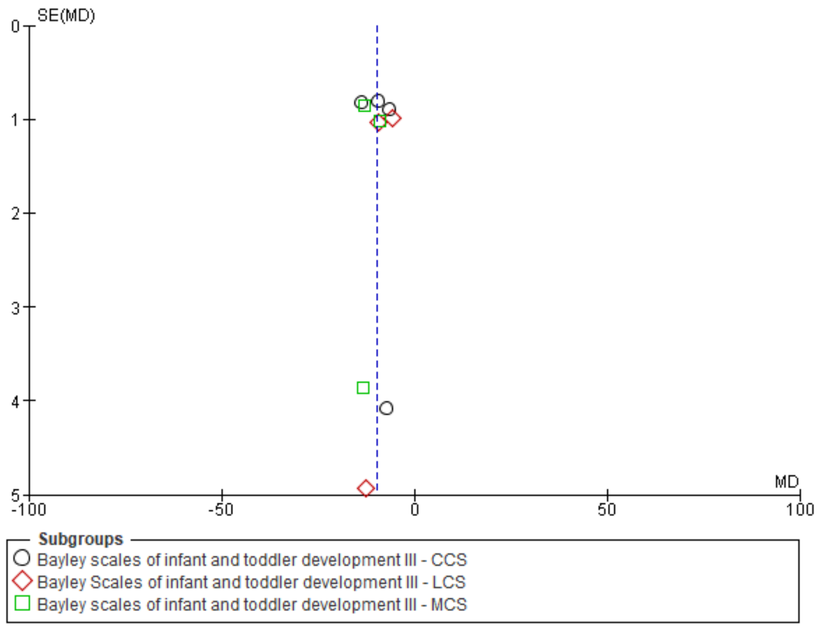

Figure S3. Funnel plot of BSID III.

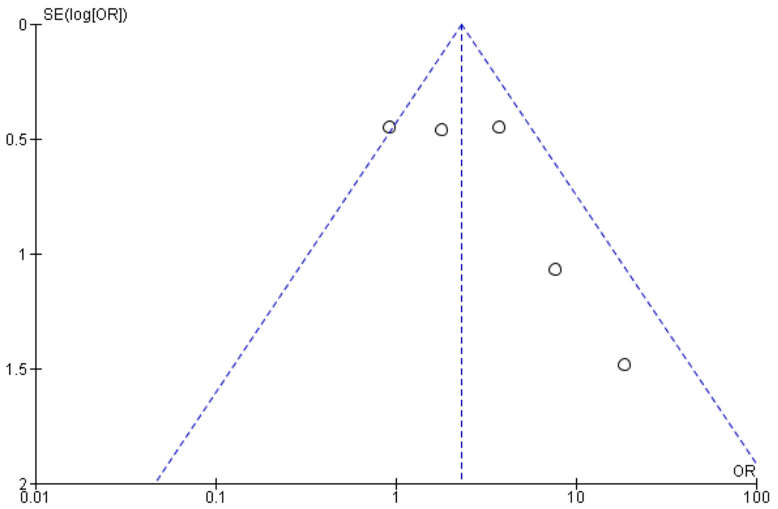

Figure S4. Funnel plot of brain abnormalities.

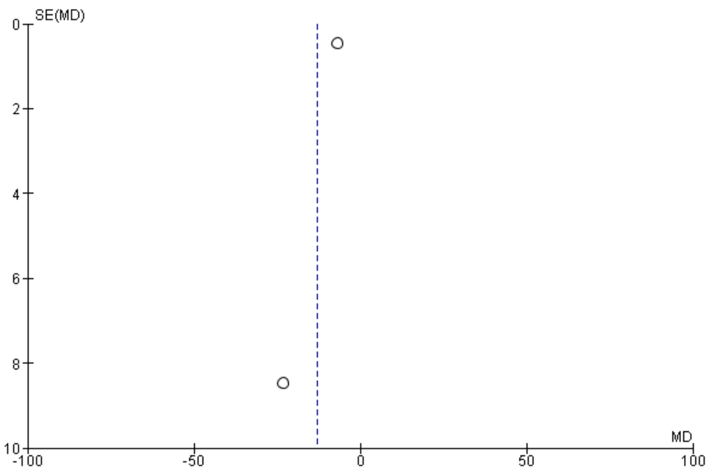

Figure S5. Funnel plot of white matter volumetry.

Appendix S4. Summary of Findings

Brain volumetry measured via MRI in preterm infants exposed to surgery compared to non-exposed preterm infants.

Patient or population: preterm infants
Setting:
Intervention: brain volumetry measured on MRI
Comparison: placebo

Outcomes	Anticipated absolute effects * (95% CI)		Relative effect (95% CI)	№ of participants (studies)	Certainty of the evidence (GRADE)	Comments
	**preterm infants w/o surgery**	**preterm infants exposed to surgery**				
white matter	The mean white matter was **0**	MD **12.96 lower** (28.35 lower to 2.42 higher)	-	259 (2 non-randomized studies)	⊕◯◯◯ Very low [a,b,c,d]	

* **The risk in the intervention group** (and its 95% confidence interval) is based on the assumed risk in the comparison group and the **relative effect** of the intervention (and its 95% CI).
CI: confidence interval; **MD:** mean difference

GRADE working group grades of evidence
High certainty: We are very confident that the true effect lies close to that of the estimate of the effect.
Moderate certainty: We are moderately confident in the effect estimate; the true effect is likely to be close to the estimate of the effect, but there is a possibility that it is substantially different.
Low certainty: Our confidence in the effect estimate is limited: the true effect may be substantially different from the estimate of the effect.
Very low certainty: We have very little confidence in the effect estimate: the true effect is likely to be substantially different from the estimate of effect.

Explanations

a. Included studies have a high risk of bias due to residual confounding factors.

b. Inconsistency was rated as serious due to the very low sample size of the included studies.

c. Heterogeneity was high for this outcome.

d. The funnel plot showed asymmetry, indicating publication bias for studies with positive results.

Brain lesions measured with MRI in preterm infants exposed to surgery compared to those with no surgical exposure.

Patient or population: preterm infants
Setting:
Intervention: brain lesions measured with MRI
Comparison: placebo

Outcomes	Anticipated absolute effects* (95% CI)		Relative effect (95% CI)	№ of participants (studies)	Certainty of the evidence (GRADE)	Comments
	**preterm infants w/o surgery**	**preterm infants exposed to surgery**				
Brain abnormalities	240 per 1.000	**389 per 1.000** (282 to 507)	**OR 2.01** (1.24 to 3.25)	528 (4 non-randomized studies)	⊕◯◯◯ Very low [a,b,c]	

* **The risk in the intervention group** (and its 95% confidence interval) is based on the assumed risk in the comparison group and the **relative effect** of the intervention (and its 95% CI).

CI: confidence interval; **OR:** odds ratio

GRADE working group grades of evidence
High certainty: We are very confident that the true effect lies close to that of the estimate of the effect.
Moderate certainty: We are moderately confident in the effect estimate; the true effect is likely to be close to the estimate of the effect, but there is a possibility that it is substantially different.
Low certainty: Our confidence in the effect estimate is limited: the true effect may be substantially different from the estimate of the effect.
Very low certainty: We have very little confidence in the effect estimate: the true effect is likely to be substantially different from the estimate of effect.

Explanations

a. Included studies have a high risk of bias due to residual confounding.

b. Included studies had a small sample size. Different scoring systems were used.

c. The funnel plot showed asymmetries, indicating publication bias for studies with positive results.

Neurodevelopmental outcome compared to placebo in premature and newborn babies

Patient or population: premature and newborn babies
Setting:
Intervention: neurodevelopmental outcome
Comparison: placebo

Outcomes	Anticipated absolute effects* (95% CI)		Relative effect (95% CI)	№ of participants (studies)	Certainty of the evidence (GRADE)	Comments
	**Risk with placebo**	**Risk with Neurodevelopmental outcome**				
Bayley Scales of Infant and Toddler Development II—BSID II cognitive score (MDI)	The mean Bayley Scales of Infant and Toddler Development II—BSID II cognitive score (MDI) was **0**a.	MD **10.98 lower** (12.04 lower to 9.91 lower)	-	9371 (6 non-randomized studies)	⨁◯◯◯ Very low [a,b,c]	
Bayley Scales of Infant and Toddler Development II—BSID II motor score (PDI)	The mean Bayley Scales of Infant and Toddler Development II—BSID II motor score (PDI) was **0.**	MD **12.98 lower** (14.08 lower to 11.88 lower)	-	9318 (6 non-randomized studies)	⨁◯◯◯ Very low [a,b,c]	
Bayley Scales of Infant and Toddler Development III—BSID III cognitive score (CCS)	The mean Bayley Scales of Infant and Toddler Development III—BSID III cognitive score (CCS) was **0.**	MD **10.11 lower** (11.06 lower to 9.16 lower)	-	7245 (3 non-randomized studies)	⨁◯◯◯ Very low [a,b,c]	
Bayley Scales of Infant and Toddler Development III—BSID III motor score (MCS)	The mean Bayley Scales of Infant and Toddler Development III—BSID III motor score (MCS) was **0.**	MD **11.55 lower** (12.83 lower to 10.27 lower)	-	4959 (2 non-randomized studies)	⨁◯◯◯ Very low [a,b,c]	

Bayley Scales of Infant and Toddler Development III—BSID III language score (LCS)	The mean Bayley Scales of Infant and Toddler Development III—BSID III language score (LCS) was **0.**	MD **7.47 lower** (8.87 lower to 6.07 lower)	-	4993 (2 non-randomized studies)	⊕◯◯◯ Very low [a,b,c]
BSID subgroup analysis in neonates with NEC—BSID cognitive score (MDI)	The mean BSID subgroup analysis in neonates with NEC—BSID cognitive score (MDI) was **0.**	MD **6.93 lower** (10.2 lower to 3.66 lower)	-	349 (4 non-randomized studies)	⊕◯◯◯ Very low [a,c,d]
BSID subgroup analysis in neonates with NEC—BSID motor score (PDI)	The mean BSID subgroup analysis in neonates with NEC—BSID motor score (PDI) was **0.**	MD **11.31 lower** (14.84 lower to 7.79 lower)	-	349 (4 non-randomized studies)	⊕◯◯◯ Very low [a,c,d]

* **The risk in the intervention group** (and its 95% confidence interval) is based on the assumed risk in the comparison group and the **relative effect** of the intervention (and its 95% CI).

CI: confidence interval; **MD:** mean difference

GRADE working group grades of evidence
High certainty: We are very confident that the true effect lies close to that of the estimate of the effect.
Moderate certainty: We are moderately confident in the effect estimate: the true effect is likely to be close to the estimate of the effect, but there is a possibility that it is substantially different.
Low certainty: Our confidence in the effect estimate is limited: the true effect may be substantially different from the estimate of the effect.
Very low certainty: We have very little confidence in the effect estimate: the true effect is likely to be substantially different from the estimate of effect.

Explanations

a. Included studies have a high risk of bias due to residual confounding.

b. Included studies had a small sample size. Different scoring systems were used.

c. The funnel plot showed asymmetries, indicating publication bias for studies with positive results.

Appendix S5. PRISMA 2020 Checklist.

Section and Topic	Item #	Checklist item	Location Where Item is Reported
**TITLE**			
Title	1	Identify the report as a systematic review.	see title
**ABSTRACT**			
Abstract	2	See the PRISMA 2020 for Abstracts checklist.	see abstract
**INTRODUCTION**			
Rationale	3	Describe the rationale for the review in the context of existing knowledge.	1 background
Objectives	4	Provide an explicit statement of the objective(s) or question(s) the review addresses.	1 background
**METHODS**			
Eligibility criteria	5	Specify the inclusion and exclusion criteria for the review and how studies were grouped for the syntheses.	2.2. Study Selection
Information sources	6	Specify all databases, registers, websites, organizations, reference lists, and other sources searched or consulted to identify studies. Specify the date when each source was last searched or consulted.	2.1. Systematic literature search
Search strategy	7	Present the full search strategies for all databases, registers, and websites, including any filters and limits used.	2.1. Systematic literature search
Selection process	8	Specify the methods used to decide whether a study met the inclusion criteria of the review, including how many reviewers screened each record and each report retrieved, whether they worked independently, and if applicable, details of automation tools used in the process.	2.2. Study Selection
Data collection process	9	Specify the methods used to collect data from reports, including how many reviewers collected data from each report, whether they worked independently, any processes for obtaining or confirming data from study investigators, and if applicable, details of automation tools used in the process.	2.2. Study Selection
Data items	10a	List and define all outcomes for which data were sought. Specify whether all results that were compatible with each outcome domain in each study were sought (e.g., for all measures, time points, analyses), and if not, the methods used to decide which results to collect.	2.3. Data Extraction
	10b	List and define all other variables for which data were sought (e.g., participant and intervention characteristics, funding sources). Describe any assumptions made about any missing or unclear information.	2.3. Data Extraction
Study risk of bias assessment	11	Specify the methods used to assess risk of bias in the included studies, including details of the tool(s) used, how many reviewers assessed each study and whether they worked independently, and if applicable, details of automation tools used in the process.	2.3. Data Extraction
Effect measures	12	Specify for each outcome the effect measure(s) (e.g., risk ratio, mean difference) used in the synthesis or presentation of results.	2.3. Data Extraction

Synthesis methods	13a	Describe the processes used to decide which studies were eligible for each synthesis (e.g., tabulating the study intervention characteristics and comparing against the planned groups for each synthesis (item #5)).	2.3. Data Extraction
	13b	Describe any methods required to prepare the data for presentation or synthesis, such as handling of missing summary statistics, or data conversions.	2.5. Statistical Analysis
	13c	Describe any methods used to tabulate or visually display results of individual studies and syntheses.	2.5. Statistical Analysis
	13d	Describe any methods used to synthesize results and provide a rationale for the choice(s). If meta-analysis was performed, describe the model(s), method(s) to identify the presence and extent of statistical heterogeneity, and software package(s) used.	2.5. Statistical Analysis
	13e	Describe any methods used to explore possible causes of heterogeneity among study results (e.g., subgroup analysis, meta-regression).	2.5. Statistical Analysis
	13f	Describe any sensitivity analyses conducted to assess robustness of the synthesized results.	2.5. Statistical Analysis
Reporting bias assessment	14	Describe any methods used to assess risk of bias due to missing results in a synthesis (arising from reporting biases).	2.4. Quality Assessment
Certainty assessment	15	Describe any methods used to assess certainty (or confidence) in the body of evidence for an outcome.	2.4. Quality Assessment
**RESULTS**			
Study selection	16a	Describe the results of the search and selection process, from the number of records identified in the search to the number of studies included in the review, ideally using a flow diagram.	3.1. Identification of Relevant Studies
	16b	Cite studies that might appear to meet the inclusion criteria, but which were excluded, and explain why they were excluded.	3.1. Identification of Relevant Studies
Study characteristics	17	Cite each included study and present its characteristics.	3.1. Identification of Relevant Studies, Table 1.
Risk of bias in studies	18	Present assessments of risk of bias for each included study.	3.3. Quality Assessment, Appendix A2.
Results of individual studies	19	For all outcomes, present, for each study: (a) summary statistics for each group (where appropriate) and (b) an effect estimate and its precision (e.g., confidence/credible interval), ideally using structured tables or plots.	3.4.-3.7.

Results of syntheses	20a	For each synthesis, briefly summarize the characteristics and risk of bias among contributing studies.	3.4.-3.7.
	20b	Present results of all statistical syntheses conducted. If meta-analysis was carried out, present for each the summary estimate and its precision (e.g., confidence/credible interval) and measures of statistical heterogeneity. If comparing groups, describe the direction of the effect.	3.4.-3.7.
	20c	Present results of all investigations of possible causes of heterogeneity among study results.	3.4.-3.7.
	20d	Present results of all sensitivity analyses conducted to assess the robustness of the synthesized results.	3.4.-3.7., Appendix A4.
Reporting biases	21	Present assessments of risk of bias due to missing results (arising from reporting biases) for each synthesis assessed.	3.3. Quality Assessment, Appendix A3, 3.8. Publication Bias
Certainty of evidence	22	Present assessments of certainty (or confidence) in the body of evidence for each outcome assessed.	3.4.-3.7., Appendix A4.
**DISCUSSION**			
Discussion	23a	Provide a general interpretation of the results in the context of other evidence.	4. Discussion
	23b	Discuss any limitations of the evidence included in the review.	4. Discussion
	23c	Discuss any limitations of the review processes used.	4. Discussion
	23d	Discuss implications of the results for practice, policy, and future research.	4. Discussion
**OTHER INFORMATION**			
Registration and protocol	24a	Provide registration information for the review, including register name and registration number, or state that the review was not registered.	5. Conclusions and Future Perspectives
	24b	Indicate where the review protocol can be accessed, or state that a protocol was not prepared.	5. Conclusions and Future Perspectives
	24c	Describe and explain any amendments to information provided at registration or in the protocol.	5. Conclusions and Future Perspectives
Support	25	Describe sources of financial or non-financial support for the review and the role of the funders or sponsors in the review.	5. Conclusions and

			Future Perspectives
Competing interests	26	Declare any competing interests of review authors.	5. Conclusions and Future Perspectives
Availability of data, code and other materials	27	Report which of the following are publicly available and where they can be found: template data collection forms; data extracted from included studies; data used for all analyses; analytic code; or any other materials used in the review.	5. Conclusions and Future Perspectives
